# Cytokinins: Wide-Spread Signaling Hormones from Plants to Humans with High Medical Potential

**DOI:** 10.3390/nu14071495

**Published:** 2022-04-02

**Authors:** Moustafa Fathy, Sahar M. Saad Eldin, Muhammad Naseem, Thomas Dandekar, Eman M. Othman

**Affiliations:** 1 Department of Biochemistry, Faculty of Pharmacy, Minia University, Minia 61519, Egypt; moustafa_fathyy@yahoo.com (M.F.); saharm1967@yahoo.com (S.M.S.E.); 2 Department of Life and Environmental Sciences, College of Natural and Health Sciences, Zayed University, Abu Dhabi 144534, United Arab Emirates; muhammad.naseem@zu.ac.ae; 3 Department of Bioinformatics, Biocenter, University of Wuerzburg, Am Hubland, 97074 Wuerzburg, Germany

**Keywords:** cytokinins, phytohormones, biological activities, plant system, mammalian system

## Abstract

Nature is a rich source of biologically active novel compounds. Sixty years ago, the plant hormones cytokinins were first discovered. These play a major role in cell division and cell differentiation. They affect organogenesis in plant tissue cultures and contribute to many other physiological and developmental processes in plants. Consequently, the effect of cytokinins on mammalian cells has caught the attention of researchers. Many reports on the contribution and potential of cytokinins in the therapy of different human diseases and pathophysiological conditions have been published and are reviewed here. We compare cytokinin effects and pathways in plants and mammalian systems and highlight the most important biological activities. We present the strong profile of the biological actions of cytokinins and their possible therapeutic applications.

## 1. Cytokinins, Occurrence, Structure and Identification

Nature is the biggest source of biologically active novel compounds. For many years, natural products have played an essential role in the management of various disorders [1,2,3,4,5,6] and constitute a large part of current-day pharmaceutical products, especially in the field of antibiotic and anticancer drugs. It is important to mention that more than 60% of current anticancer drugs come from natural sources [7,8,9,10]. In other pathophysiological conditions, such as cardiovascular diseases, diabetes mellitus, and multiple sclerosis, natural products have contributed clearly to the therapeutic protocols [11,12,13,14]. Some of the recently highlighted natural products are cytokinins (CKs), which have different pharmacological and medicinal actions. 

Investigation of cytokinins started in 1948, when Caplin and Steward [15] found that coconut milk positively affects the growth of the carrot root, indicating the presence of a compound that affects the cell proliferation and division. CKs were discovered during the researchers’ efforts to detect some specific inducers for cell division [16]. These investigations encouraged Skoog, Miller, and co-workers in 1955 [17] to isolate and identify Kinetin, a highly active cell-division stimulator, from autoclaved herring sperm DNA. In the search for other naturally occurring CKs in plant tissues, Letham in 1963 [18] isolated zeatin, a “kinetin-like” factor, from immature corn kernels.

After many reports, plant hormones, or phytohormones, were defined as the family of small molecules and plant-specific signaling messengers found in higher plants, mainly in the developing fruits, xylem sap, shoot apex, root tips, immature seeds, and tumor tissues. We now know that the occurrence of CKs extends to algae, yeasts, bacteria, insects, mosses, and mammals [19,20,21,22,23,24], and recently, CKs have been identified in various dog tissues mammalian cells and urine [25,26,27]. In addition, various plant-interacting microbes—different types of pathogenic and non-pathogenic bacteria, some fungi and animal pathogens such as mycobacterium tuberculosis—have also been shown to produce CKs [28,29,30].

**Figure 1:**
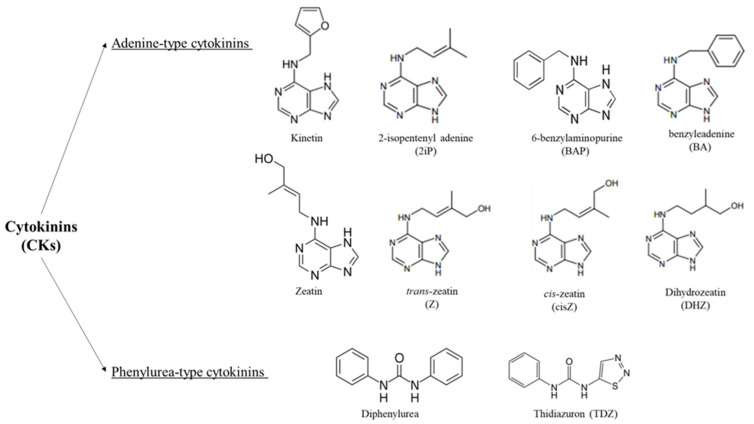
Chemical structures of cytokinins (CKs); adenine-type and phenylurea-type cytokinins are shown.

Structurally, CKs are adenine derivatives substituted with their isoprenoid or aromatic side chain at the N6-position (Figure 1). Isoprenoid cytokinins are present in all plants, whereas aromatic CKs with N6-benzyl substituents have only been found in certain taxa [31,32], there are two types of CKs: adenine-type CKs represented by kinetin, zeatin, and 6-benzylaminopurine, and phenylurea-type CKs such as diphenylurea and thidiazuron (TDZ) [33]. For the activation of CKs, cytokinin riboside 5′-monophosphate phosphoribohydrolases are required, in which this enzyme converts inactive cytokinin nucleotides directly into the active free bases and regulating the actions, biosynthesis, and activity of CKs [34,35,36,37,38]. 

The presence of CKs in DNA and cell extracts encouraged researchers to study the mechanism of their formation at the nucleic acid level [39]; for instance, kinetin, the first identified and most common CK, has an N6-furfuryl side chain. Barciscwski et al. [40,41] proposed that endogenous kinetin is produced as a result of oxidative DNA damage. Interestingly, the chemical structure of kinetin showed that the formation of its molecule may be the result of the reaction of an adenine residue of DNA with furfural [41], and recently, furfural was reported to be formed during oxidative damage to DNA in vitro whereas another intramolecular rearrangement yields kinetin in vivo [42]. In addition, kinetin was found to be the precursor to kinetin triphosphate (KTP), an ATP analog produced upon the salvaging of kinetin by adenine phosphoribosyltransferase (APRT) [43]; such promising findings led to the investment of more efforts into understanding the cytokinin biosynthesis pathways [44,45,46,47].

Different analytical methods were reported for the identification of CKs in their diverse sources such as immunological methods, gas chromatography–mass spectrometry (GC-MS), Sephadex LH-20 column chromatography, and bioassays [22,24,25,48,49].

Certain pharmaceutical studies investigated different approaches to increase the efficacy of kinetin and its pharmacokinetics; for example, it was suggested that to increase its absorption, kinetin could be added into a solid–lipid nanoparticle [50] or liposome [51] or mixed with other bioactive compounds [51,52].

## 2. Cytokinin Action in Plants

CKs play a major role in plants, both over long distances (paracrine signaling) and on the cell that produced them (autocrine signaling). They participate in almost every aspect of plant biology, including cell division and differentiation, organogenesis, alleviation of different biotic and abiotic stresses, and many other physiological and developmental processes [53,54,55]. The type and activity of CK molecules differ remarkably between different plant species and tissues, at different developmental stages, under various environmental conditions such as low night temperature, and interference of other factors such as the amount of melatonin and the expression of CK receptors [56,57,58,59]. 

Mechanistically, the CK signaling pathway in plants is the same as in bacteria and yeast: CKs trigger receptor histidine kinases as part of the his-asp-phosphorelay system, resembling the bacterial two-component system of histidine phosphotransfer protein and response regulator [60]. This eukaryotic two-component system (TCS) is unique in plants among higher eukaryotes [61,62].

Skoog and Miller (1957) [63] revealed that undifferentiated callus cultures would be differentiated into roots or shoots depending on the relative amount and ratio of CK and auxin in the medium; interestingly, a balanced ratio keeps the cells in an undifferentiated state, while high CK to auxin ratios promote shoot development and low ratios promote root development.

Several studies reported the detailed action of CKs in plants, highlighting their role in inducing the immunity of plants against different pathogens [30,53,54]. For instance, ethylene-cytokinin participated in the defense response of wheat against *Stagonospora nodorum* Berk, where exogenous application of zeatin improved the wheat resistance against *S. nodorum* through attenuation of the ethylene signaling pathway and up-regulation of salicylic acid (SA)-dependent genes [64], whereas Gupta et al. [65] reported that CK response regulates the systemic immune response and fungal pathogen resistance by modulating the trafficking of the PRR LeEIX2 in the tomato. This mediates the immune response to Xyn11 family xylanases and promotes resistance to *Botrytis cinerea* and *Oidium neolycopersici* by SA- and ethylene (ET)-dependent mechanisms [65]. In another study, CKs have been shown to mediate a defense against insecticide toxicity, e.g., against cypermethrin insecticide toxicity in the cyanobacterium *Nostoc muscorum*, by involving nitric oxide, regulation of exopolysaccharide secretion, and reactive oxygen species (ROS) homeostasis [66].

In a recent study, it was found that CK signaling is a potential target for enhancing future shoot regeneration efficiency, as it activates the dedication of the shoot progenitor at later stages and allows chromatin to maintain shoot identity genes at the priming stage [67]. In the context of the plant growth phases, it is frequently reported that CKs have an important regulatory role, for instance, vegetative phase change in *Arabidopsis thaliana* through the miR172/TOE1-TOE2 module [68], development and environmental responses of plants through CKRs [69], and shoot branching regulation in *Pisum sativum* through the SMXL/D53 strigolactone signaling repressors and up-regulation of PsSMXL7/D53 transcripts [70]. 

In addition to the direct action of the CKs on plant organogenesis and growth, they also interfere with the level of phenolic and flavonoids compounds, as it was found that the antioxidant and cytotoxic effects in regenerated red cabbage were enhanced by the application of zeatin [71].

Furthermore, it was reported that 6-benzylaminopurine (BAP), which is a growth-regulatory plant cytokinin, could alleviate the detrimental effects of copper-induced toxicity in cotyledonal leaves of *R. communis* by effectively modulating stomatal responses and antioxidation mechanisms, thereby enhancing the function of the photosynthetic apparatus [72]. In addition, 2-isopentenyl adenine (2iP) and benzyleadenine (BA) increased the production of organogenic nodular meristemoids and the regeneration of *Tylophora indica* roots [73]. Additionally, BA was observed to increase the growth of shoots in *Gynura procumbens* (Lour.) Merr, which is a medicinal plant that has antioxidant, anticancer, anti-inflammatory, hepatoprotective, and antimicrobial activities [74]. Furthermore, BA was found to improve the antioxidant enzyme activities and total antioxidant capacity and maintain higher levels of ATP content and energy charge in plants [75].

## 3. Cytokinins in Mammals

Knowledge of CKs’ action in plants and their protective activities encouraged researchers to focus on their potential effects in mammals and their utility for treating human diseases or serving as prophylactic agents, in addition to the investigation of the responsible signaling mechanisms and pathways [52,76,77,78,79,80]. Many studies have been conducted to examine the various pharmacological activities of natural phytohormones, CKs, and many authors reported that CKs have wide range of pharmacologic and health-promoting properties including neuroprotective, immunomodulatory, and anti-proliferative effects, in addition to numerous prospective therapeutic applications [81,82,83].

## 4. Antioxidant Activity of Cytokinins

Antioxidant activity is one of the highly reported biological activities for CKs. Since 1996, kinetin has been reported to have an antioxidant effect. It could control the level of ROS in the cell by direct clean up via different mechanisms, such as the presence of a Furan ring, which is rich in electrons and acts as a scavenger of ROS [84,85]; the reaction with copper to produce complexes, which have superoxide-dismutase (SOD)-like action [86]; or the interaction of kinetin with iron, causing a reduction in 8-hydroxy-2-oxyguanosine production [87]. Another reported mechanism is the prevention of routine oxidation and glycoxidation formation and prevention of the formation of advanced glycation age products and protein-aggregation-induced fragmentation [88].

In 2016, our group reported that kinetin exerts antioxidant activity and an antigenotoxic effect in different mammalian cell lines representing different organs. In addition, we highlighted that CKs could have dual actions according to the applied concentration [89], and our results were supported by another study, which showed the antioxidant activity as a mechanism for the antigenotoxic effect of kinetin [90].

Bizzalori chose four CKs to evaluate their antioxidant activity by using a group of different fluorescent and spectrophotometric assays. Kinetin was demonstrated to have the highest antioxidant activity up to a concentration of 1 µM. The author suggested that some of the biological activity of the tested CKs is due to an intrinsic antioxidant capacity [91].

In cultured astrocytes and mouse brain, kinetin showed protection against both D-galactose-induced oxidative damage and enhanced cell viability through attenuation of the antioxidant system super oxide dismutase (T-SOD), glutathione peroxidase (GSH-PX), and malonyl dialdehyde concentration in the cell membrane [81].

In another study, where cognitive impairment and oxidative damage were induced in mouse models by aluminum chloride and D-galactose, kinetin had the ability to enhance the antioxidant system of the cell by increasing the activities of SOD, GSH-px, and heme oxygenase 1 (HO-1) [92]. In addition, free radical scavenging activity of kinetin was evaluated in vitro in inactivated platelets, where the results proved that kinetin suppressed hydroxyl radical formation in a dose-dependent manner and exerted antithrombotic activity in three in vivo models as well [93].

The antioxidant activity of kinetin was extended to preserve the structural and functional integrity of dog sperm during cryopreservation, where kinetin-supplemented samples were characterized by higher sperm counts with intact plasma membrane, normal acrosomes, mitochondria, and chromatin, and the samples showed a significant increase in the expression levels of anti-apoptotic (*BCL2*) and *protamine-related* (*protamine* 2, *PRM2*; *protamine* 3, *PRM3*) genes and a decrease in the expression of pro-apoptotic (*BAX*) and mitochondrial *reactive oxygen species-modulating* (*ROS modulator* 1, *ROMO1*) genes [94]. In the same direction, in 2018, a research group examined the ability of kinetin to preserve the quality of ram semen during storage at refrigerator temperature. At the end of the study, they concluded that kinetin improved spermatozoa motility and viability, the function of the spermatozoa plasma membrane, and the antioxidative markers of the ram semen [95].

## 5. Anti-Aging Activity of Cytokinins

Anti-aging activity is a well-known pharmacological action of CKs in human tissues. In vitro, trans-zeatin and kinetin showed the same effect on aging markers of fibroblasts; both compounds delayed the onset of several cellular and biochemical markers that characterize the cellular aging of cultivated fibroblasts [96,97], where the treatment of keratinocytes with a combination of high levels of calcium and kinetin increased the expression of different markers of cell differentiation, and treatment of fibroblasts with kinetin or N6-benzyladenine affected the antioxidative enzyme activity, reduced glutathione and thiol group content, decreased the membrane phospholipid peroxidation, and exhibited protective properties against malondialdehyde production [98,99,100]. 

In a study with primary keratinocyte cultures of psoriatic patients, it was observed that kinetin stimulated the differentiation of the psoriatic cells and modified their hyper-proliferative activity, which induced high numbers of cells to develop cornified envelopes [101]. McDaniel et al. examined the protective effect of kinetin against UVB radiation in keratinocytes: a 36% reduction in thymine dimer formation in DNA was observed in cells treated with 100 µM kinetin and exposed to UVB [102]. Another study investigated the effect of trans-zeatin on UVB-induced matrix metalloproteinase (MMP) expression in human skin fibroblasts. It was found that trans-zeatin (20–80 µM) markedly down-regulated UVB-induced MMP expression in a dose-dependent manner, resulting in enhanced cell viability of the radiated fibroblasts [103].

Ji et al. tested the anti-aging protective effect of trans-zeatin in the immortalized HaCaT keratinocytes against UV irradiation. Trans-zeatin induced aquaporin-3 (AQP3) expression and attenuated the UV-induced loss of AQP3, and UV-induced decreased water permeability in HaCaT cells, in addition to the inhibition of the activation of the UV-induced MEK/ERK pathway, which plays an important role in UV-induced AQP3 loss [104]. 

Topical applications of CKs for skin care purposes have been widely studied. The safety and efficacy of products containing kinetin have been investigated in many studies on animal models and human volunteers. Different studies found that kinetin, or its derivatives, which were tolerated in those studies, significantly improved the symptoms of rosacea and photo-damaged skin, such as facial skin erythema, skin texture, roughness and moisture, mottled hyperpigmentation, and fine wrinkles, even within 2 to 12 weeks of treatment [52,102,105,106,107,108,109]. In another study, a trial extended for 48 weeks of application in 18 subjects, resulting in a 44% reduction in erythema severity and an 89% reduction in inflammatory lesions. This long-term treatment exhibited the absence of skin irritation, which is an important advantage, as skin sensitivity is a common side effect in patients with rosacea treated with long-term topical cosmetics [110].

Kimura and Doi conducted a study with a long-term topical application of kinetin in 10-year-old hairless dogs; the model was characterized by age-related changes in the skin similar to those in humans. Obvious improvements in skin texture, reduced wrinkling and pigmentation, decrease in the thickness of the corneal layer, a smaller number of melanin granules, and increased numbers of collagen and elastic fibers in the dermis were observed after 100 days of application, even with lower concentration. It is important to mention that no side effects were observed, indicating the safety of using kinetin for long-term therapy [111]. 

The anti-aging activity of CKs was also investigated regarding skeletal muscle aging. In a recent study [112], it was reported that kinetin efficiently stimulates in vitro differentiation of C2C12 myoblasts into myotubes. This action was explained by its antioxidant activity and its ability to modulate intracellular calcium levels [112]. Moreover, kinetin reduced coronary atherosclerosis by its ability to improve the LDL/HDL ratio [113,114].

## 6. Anticancer Activity of Cytokinins

A long time ago, the anticancer activity of CKs caught the attention of the scientists: in 1960, an in vitro study was conducted to examine the effect of kinetin and kinetin ribofuranoside on tissue cultures of human skin and breast carcinoma. This showed antiproliferative activity against the outgrowth of both tissues [115]. Further reports about natural CK ribosides (iPR, KP, BAR, and OTR) showed that they had high toxicity to cancer cell lines. Instead, their corresponding nucleotide base had no or limited toxicity [116]. 

In contrast to human cells, plant cells can convert both forms of CK (cytokinin bases and their corresponding ribosides) riboside-5-mono phosphatase; this explains the low toxicity of kinetin in human leukemia HL-60 cell line, which is due to the activity of human phosphoribosyl transferase toward cytokinin bases [77]. Accordingly, it was reported that different structural requirements are essential for CKs to exhibit their cytotoxic activity against human cell lines that differ from those required in plant bioassays. The ribose moiety appears to be important for a cytotoxic effect in human cells [77]. In addition, the hydroxyl position of the side chain of kinetin had a marked effect on anti-cancer activity in both aromatic (oTR >> mTR, pTR) and isoprenoid CKs.

In 2010, Voller et al. confirmed the cytotoxic activity of N6-isopentyl adenosine, kinetin riboside, and N6-benzyl adenosine and extended the scope of their toxic effect to a wider range of cancer cell lines. In that study, the authors provided the first evidence of cytotoxic activity of the hydroxylated aromatic CKs (ortho, meta, para topoline riboside) and the isoprenoid cytokinin cis-zeatin riboside. Another significant finding showed that cytokinin-free bases (2-methylthioderivatives as well as O- and N- glucoside) showed no or limited toxicity [116].

In 2017, Vollar et al. conducted a study to compare the activity of kinetin riboside N6-benzyladenosine (BAR) and N6-isopentenyladenosine with those of the highly active, naturally occurring aromatic cytokinin OTR (ortho topoline riboside) and 20H3MeoBAR N6-(2-hydroxy-3-methoxybenzyl) adenosine. The authors reported that 20H3MeoBAR is the most effective studied cytokinin riboside because it can cause its effect by stimulating cell death without ATP depletion. This study also includes NCI-60 cytotoxicity assay, which demonstrated that the activity of 20H3MeoBAR is independent of p53 status [117].

In leukemia cell lines, CK bases do not exhibit any significant cytotoxic effect; however, they can cause differentiation in some specific types of leukemia cell lines. The differentiation therapy is characterized by a lesser side-effect compared to other regimens, which lead to cell death. Kinetin and other cytokinin bases, including kinetin, N6-isopentenyladenine, and N6-benzyladenine, at concentrations above 25 µM induce granulocytic differentiation in the HL-60 cell line [118,119], and the phosphorylation of ERK1/2 and expression of S100P and CEBPD proteins are reported as suggested pathways through which differentiation takes place [120]; however, low micromolar concentrations of iPR-induced rapid apoptosis in HL-60. Combination treatment of cytokinin ribosides with caspase inhibitors shifted the cytokinin riboside activity from pro-apoptotic to growth-inhibitory and differentiating [118]. Therefore, we can say that differentiation (possibly occurring at low concentrations) could contribute to the therapeutic effects of CKRs in myeloproliferative diseases. Cytokinin bases are also able to promote differentiation of keratinocytes [98] and could have a potential role in psoriasis therapy.

Interference with mitochondrial functions and potential antimitotic effects of kinetin riboside were reported as suggested mechanisms for their anticancer activity [121]. This is supported by metabolism in cancer cells, which mainly occurs through the Crabtree effect and relies on glucose-induced inhibition of cell respiration and oxidative phosphorylation (OXPHOS), supporting the survival of cancer cells under metabolic stress conditions. Technically, replacing glucose with galactose in the culture changes the cells to make them more sensitive to mitochondrial perturbations caused by antimitotic toxins. Kinetin riboside in the galactose environment was found to be a potent apoptosis-inducing agent, decreased the mitochondrial membrane potential, reduced glutathione level, depleted cellular ATP, and induced ROS production in the OXPHOS state, leading to a loss of cell viability [121]. In another study, it was reported that kinetin riboside induces apoptosis in HeLa and mouse melanoma B16F-10 cells by modulating the mitochondrial membrane potential, stimulating the release of cytochrome c and activating caspase-3. In contrast, human skin fibroblast CCL-116 and bovine primary fibroblast cells showed resistance to kinetin and no significant changes in Bad, Bcl-X(L), and cleaved PARP were observed [122]. In HSCs cells, the kinetin-induced apoptosis was positively correlated with the expression of Bax and negatively with the expression of Bcl-2. Moreover, kinetin inhibited the growth of HSCs by interrupting the cell cycle at the G1/S restriction point and inducing apoptosis by reducing the Bcl-2/Bax ratio [123].

In human prostate cancer cells, kinetin riboside inhibited the growth of cancer cells and affected the expression of different proteins such as N-cadherins, Vimentin, Snail, Twist, p-Akt, antiapoptotic Bcl-2, Bax, and MMPs. It was found that this effect was dependent upon the type of the cells and their androgen sensitivity [124].

The combination of cytokinin ortho-methoxytopolin-riboside (MeoTR) and auxin indole-3-acetic acid (IAA) shows synergistic anticancer activity in HeLa cells by inducing apoptosis, cell cycle progression arrest in S phase, and blockage of the Akt pathway [125]. 

Crude extracts and purified cytokinin fractions from the mycelia of medicinal mushrooms Hericium coralloides and Fomitopsis officinalis were examined for their effect on the growth and morphology of HepG2 cells. The results showed that purified cytokinin induced cytotoxicity and apoptosis in the HepG2 cells [126]. In addition, it was reported that different derivatives of BAP have cyclin-dependent, kinase-inhibitory, anticancer, and antiproliferative properties on various cancer cell lines [127].

## 7. Neuroprotective Activity of Cytokinins 

Kinetin was demonstrated to control some human mRNA splicing diseases such as familial dysautonomia [128,129,130] and the serious disease Neurofibromatosis type 1 (NF1), which is a characterized by the presence of ‘café au lait’ spots and neurofibromas and associated with mutation, which may lead to malignant tumors [131].

Further, various studies reported that natural CKs have a therapeutic potential in the treatment of age-related neurodegenerative diseases as Huntington’s disease (HD) [132] and Parkinson’s disease (PD) [133] with few side effects, and explained that kinetin can act as a phosphate donor after its conversion by adenosine phosphoribosyl transferase (APRT) into its triphosphate form, maintaining the N17 phosphorylation in HD model neurons and mutant huntingtin expression cells [132].

In 2013, Hertz et al. discovered that kinetin could be a precursor of N6-modified ATP analogs K triphosphate (KTP), and in a pink1 kinase-dependent manner, it can accelerate parkin recruitment to depolarized mitochondria, block mitochondrial motility in axons, and suppress apoptosis of human neural cells [43].

The activity of CKs as neuroprotective agents is not limited to the compounds themselves but extends to their metabolites. Gonzalez et al. proved that in neuron-like SH-SY5Y cells, cis-zeatin riboside, kinetin-3-glucoside (K3G), and N6-isopentyl adenosine had a clear neuroprotective cytotoxic effect [133] and zeatin riboside exerted its potential neuroprotective effect via activation of the A2A-R signaling in mutant huntingtin Pcl12 cells [134].

In another study, kinetin attenuated the anxiety and improved the memory impairment caused by the exposure to a toxic dose of radiation [135]; it also protected immortalized hippocampal cell line (HT-22) from glutamate-induced cell death by promoting the nuclear translocation of NrF2, inducing HO-1 expression, suppressing ROS, and increasing intracellular calcium influx [136].

In a recent study focusing on familial *Dysautonomia* disease (FD), which is characterized by an autosomal recessive congenital neuropathy and impaired level of IκB kinase complex-associated protein (IKAP), a treatment protocol of a combination of kinetin with phosphatidyl serine (PS) or pridopidine increased the IKBKAP gene level with no side effects in comparison to PS or pridopidine alone [137].

In HT-22 cells, kinetin provided a neuroprotective effect, attenuated glutamate-induced oxidative cytotoxicity, and rescued cell death by suppressing the accumulation of intracellular free radicals, increasing the intracellular calcium influx, maintaining normal function of mitochondria, and activating Nrf2 pathway [138].

## 8. Anti-Inflammatory and Immunomodulatory Effects of Cytokinins 

Inflammatory and immune responses are important for the maintenance of physiological functions and repair processes of the body. In 2015, Lappas [139] showed that zeatin riboside modulated mammalian T lymphocyte and immune system activity via an A2AR-dependent mechanism and was considered as an effective therapeutic potential in the treatment of chronic inflammatory disorders and thioglycolate-induced peritoneal leukocytosis. Zeatin riboside treatment stimulated the production of cyclic adenosine monophosphate (cAMP) by T lymphocytes and suppressed the production of interferon (IFN)-c, IL-2, tumor-necrosis factor (TNF)-a, IL-4, and IL-13 by CD31CD41 T cells and the production of IFN-c, IL-2, and TNF-a by CD31CD81 T cells. In addition, zeatin riboside modulated the up-regulation of CD25, CD69, and CD40L by activated T lymphocytes [139].

In a study on PC-12 cells, it was found that zeatin prevented amyloid beta-induced neurotoxicity and scopolamine-induced cognitive deficits, and in the same study, the effect of zeatin on learning and memory capacity in vivo using ICR mice was evaluated. Zeatin showed antioxidant and cell protective effects against Abeta-induced neurotoxicity and ameliorated scopolamine-induced amnesia, suggesting a potential chemopreventive role of zeatin in Alzheimer’s disease [140]. 

Familial dysautonomia (FD) is a recessive neurodegenerative disease caused by a T to C transition at base pair 6 of IKBKAP intron 20. Hims et al. [129] reported that kinetin increased exon 20 inclusion in RNA isolated from cultured FD cells and increased IKBKAP mRNA and IKAP protein to normal levels in FD lymphoblast cell lines. Furthermore, Slaugenhaupt et al. [128] supported the idea of using kinetin for the treatment of other human splicing disorders by showing correction of a splicing defect in neurofibromatosis [128,129]. 

## 9. Conclusions

Collecting all the previous reports about the biological actions of CKs (Table 1), we can conclude that the plant hormones cytokinins are promising natural products for their pharmacological and prophylactic activities in mammalian cells, and the latest pharmacological results reviewed here underline an impressive broad potential for CKs in treating a number of diseases by inducing regeneration.

## Figures and Tables

**Table 1:** Summary of the different pharmacological activities and the responsible mechanisms for cytokinins.

Activity	Mechanism
Antioxidant [81,86,87,88,89,90,91,92,93,94,95]	Kinetin controls the level of ROS via - Scavenging the ROS (due to the presence of furan ring); - Production of complexes which have SOD-like action. Kinetin and BA protect against oxidative damage via - Attenuation of GSH-PX activity; - Decreasing the membrane phospholipid peroxidation and MDA production; - Increasing the activities of SOD and HO-1. Kinetin increases the expression levels of anti-apoptotic (*BCL2*) and *protamine-related* (*PRM2* and *PRM3*) genes and decreases the expression of pro-apoptotic (*BAX*) and mitochondrial *reactive oxygen species-modulating* (*ROMO1*) genes
Antithrombotic [93]	Kinetin suppressed hydroxyl radical formation
Anti-aging [52,96,97,98,99,100,101,102,103,104,105,106,107,108,109,110,111,112,113,114]	Kinetin - Increases the expression of different markers of cell differentiation; - Improves skin texture, reduces wrinkling and pigmentation, decreases the thickness of the corneal layer, and increases numbers of collagen and elastic fibers in the dermis; - Decreases the skeletal muscle aging by its antioxidant activity and its ability to modulate intracellular calcium levels. *trans*-zeatin - Down-regulates UVB-induced MMP expression resulting in enhanced cell viability of the radiated fibroblasts; - Modulates AQP3 expression; - Inhibits UV-induced MEK/ERK pathway activation.
Anticancer [77,98,115,116,117,118,119,120,121,122,123,124,125,126,127]	Kinetin, 2iP and BA show antiproliferative activity via - Induction of differentiation by the phosphorylation of ERK1/2 and the expression of S100P and CEBPD proteins; - Induction of apoptosis by ▪ modulating the mitochondrial membrane potential; ▪ depleting cellular ATP; ▪ stimulating the release of cytochrome c; ▪ activation of caspase-3; reducing the ratio of Bcl-2/Bax; ▪ interrupting the cell cycle at G1/S restriction point; - Inhibition of the growth of the cancer cells by modulating the expression of different proteins such as N-cadherins, Vimentin, Snail, Twist, p-Akt, and MMPs.
Neuroprotective [43,128,129,130,131,132,133,134,135,136,137,138]	Kinetin - Acts as a phosphate donor maintaining the N17 phosphorylation in Huntington’s disease; - Attenuates the anxiety and improved the memory impairment by ▪ Promoting the nuclear translocation of NrF2; ▪ Induction of HO-1 expression; ▪ Suppression of ROS; ▪ Increasing of intracellular calcium influx. Zeatin showed an potential neuroprotective effect via activation of the A2A-R signaling.
Anti-inflammatory and immunomodulatory [128,129,139,140]	Zeatin riboside modulated mammalian T lymphocyte and immune system activity via an A2AR-dependent mechanism by - Stimulation of the production of cAMP by T lymphocytes; - Suppression of the production of IFN-c, IL-2, TNF-a, IL-4 and IL-13, by CD31CD41 T cells; - Production of IFN-c, IL-2 and TNF-a by CD31CD81 T cells. Kinetin improves familial dysautonomia by increasing exon 20 inclusion in RNA and increasing *IKBKAP* mRNA and IKAP protein to normal levels.

## Data Availability

Not applicable.
